# Exploration of how primary care models influence job satisfaction among primary care providers during the COVID-19 pandemic in New Brunswick: a descriptive and comparative study

**DOI:** 10.1186/s12913-023-09211-2

**Published:** 2023-03-07

**Authors:** Claire Johnson, Dominique Bourgoin, Jérémie B. Dupuis, Jenny Manuèle Félix, Véronique LeBlanc, Danielle McLennan, Luveberthe St-Louis

**Affiliations:** School of Public Policy, Université de Moncton, Moncton, New Brunswick E1A 3E9 Canada

**Keywords:** Physician shortage, Primary care, Models of care, Primary care providers, Retention, Recruitment

## Abstract

**Background:**

The COVID-19 pandemic has highlighted human resource gaps and physician shortages in healthcare systems in New Brunswick (NB), as evidenced by multiple healthcare service interruptions. In addition, the New Brunswick Health Council gathered data from citizens on the type of primary care models (i.e. physicians in solo practice, physicians in collaborative practice, and collaborative practice with physicians and nurse practitioners) they use as their usual place of care. To add to their survey’s findings, our study aims to see how these different primary care models were associated with job satisfaction as reported by primary care providers.

**Methods:**

In total, 120 primary care providers responded to an online survey about their primary care models and job satisfaction levels. We used IBM’s “SPSS Statistics” software to run Chi-square and Fisher’s exact tests to compare job satisfaction levels between variable groups to determine if there were statistically significant variations.

**Results:**

Overall, 77% of participants declared being satisfied at work. The reported job satisfaction levels did not appear to be influenced by the primary care model. Participants reported similar job satisfaction levels regardless of if they practiced alone or in collaboration. Although 50% of primary care providers reported having symptoms of burnout and experienced a decline in job satisfaction during the COVID-19 pandemic, the primary care model was not associated with these experiences. Therefore, participants who reported burnout or a decline in job satisfaction were similar in all primary care models. Our study’s results suggest that the autonomy to choose a preferred model was important, since 45.8% of participants reported choosing their primary care models, based on preference. Proximity to family and friends and balancing work and family emerged as critical factors that influence choosing a job and staying in that job.

**Conclusion:**

Primary care providers’ staffing recruitment and retention strategies should include the factors reported as determinants in our study. Primary care models do not appear to influence job satisfaction levels, although having the autonomy to choose a preferred model was reported as highly important. Consequently, it may be counterproductive to impose specific primary care models if one aims to prioritize primary care providers’ job satisfaction and wellness.

## Background

The COVID-19 pandemic has highlighted human resource gaps in healthcare systems across Canada and elsewhere. New Brunswick’s healthcare services are also weakened by a physician shortage that may disproportionately affect rural areas [1, 2]. Across the province, there are approximately 180 vacant physician positions; most are in the more rural and remote areas [3]. Furthermore, in the next five to seven years, approximately 35% of family physicians in New Brunswick are expected to retire [4]. This physician shortage is increasingly concerning as it has already started to impact patient care, as evidenced by service interruptions in both Regional Health Authorities responsible for healthcare delivery in New Brunswick [5–7]. In addition to these long-standing challenges, the COVID-19 pandemic has increased the strain on the healthcare workforce. There are also multiple reports of physicians leaving the province for reasons that remain largely unknown [8, 9].

**Fig. 1:**
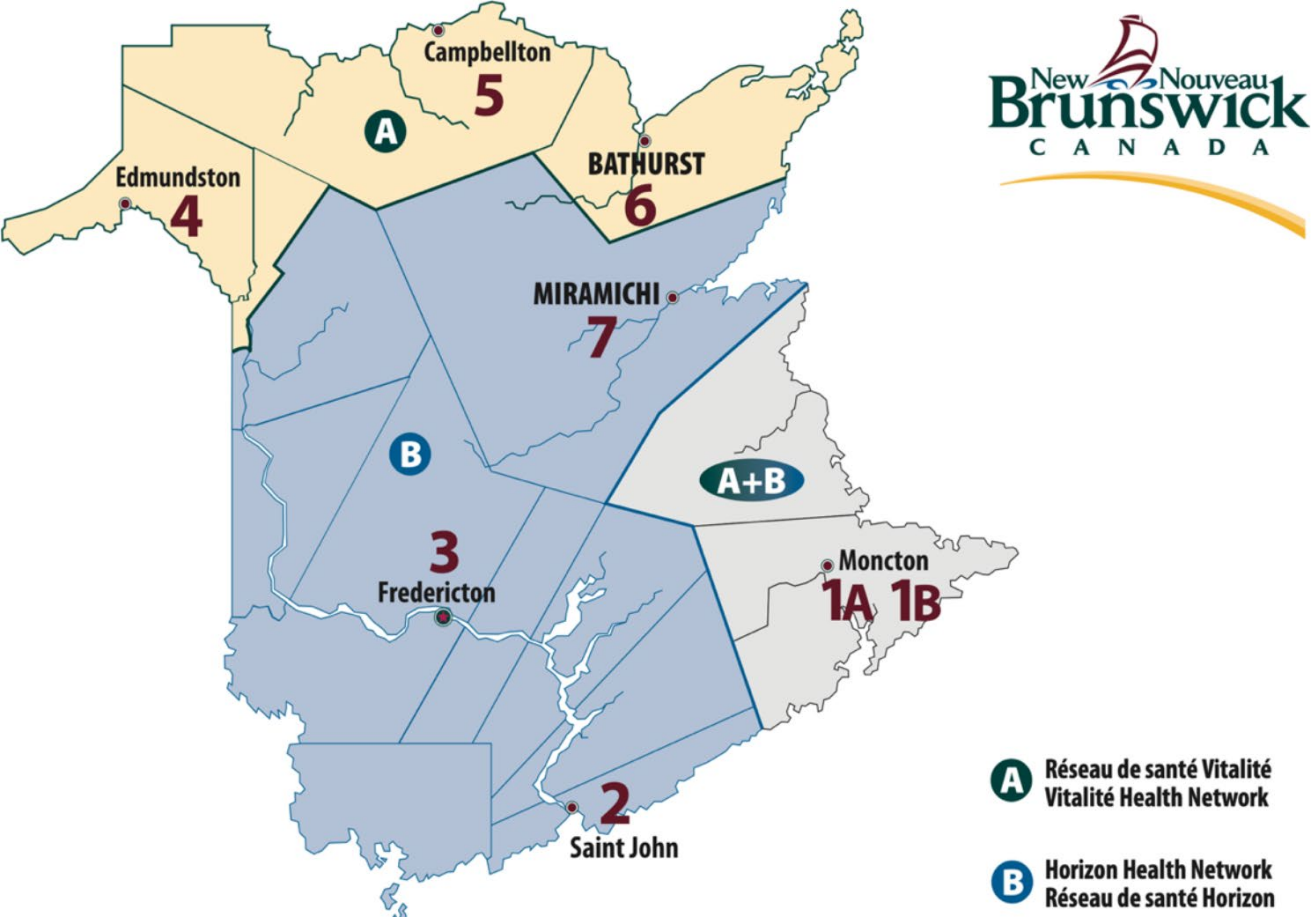
Health zones in New Brunswick [14]

Studies regarding factors affecting physician retention (looking at indicators like job satisfaction, burnout, and intentions to leave the profession or area of practice) suggests that a reasonable workload, community support, work-life balance, and effective collaborations between physicians are predictors of job satisfaction, and may reduce the likelihood of physicians wanting to leave their practice or the medical profession altogether [10, 11]. In addition, evidence shows that job satisfaction may vary depending on the practice environment [12] or the community where the physician practices [11]. A Canadian study found that job satisfaction was different for physicians practicing in a rural setting compared to physicians working in an urban setting, highlighting the benefit of trying to match physicians with practice environments and communities that suits their preferences [12]. This is relevant to the New Brunswick setting since some physicians work in rural settings, and others in urban settings throughout the various geographical zones, as shown in Fig. 1 below. Communities within 40 km of Moncton (zone 1), Saint John (zone 2) and Fredericton (zone 3) are considered urban, whereas communities outside of the 40 km range are considered rural [13]. The rural communities in the table below are Edmundston (zone 4), Campbellton (zone 5), Bathurst (zone 6) and Miramichi (zone 7).

Primary care is known to be the cornerstone and the first point of contact in most healthcare systems in developed countries [15, 16]. In New Brunswick, primary care may look different depending on the primary care model. Recently, the New Brunswick Health Council gathered data from citizens to find out what type of primary care models they reported using as their usual place of care [17, 18]. These different primary care models (family physicians, after-hours/walk-in clinics, emergency department) exist in varying proportions across New Brunswick’s health zones. Patients without a consistent primary care provider, representing 10% of the population, or approximately 80,000 citizens in NB, are individuals who are most likely to use alternate models of primary care regularly [17].

Before our study, there was little data examining primary care models from the provider’s perspective. Given the abundance of media coverage related to physician shortages in New Brunswick, it is crucial to investigate which primary care models could improve job satisfaction and retention. However, extensive media coverage contrasts with the scarcity of scientific studies on the subject. Consequently, there were knowledge gaps about the relationship between job satisfaction and various primary care models. This latest information could potentially help reduce the physician shortage in New Brunswick through more effective recruitment and retention strategies. Additionally, the information gathered from the primary care providers’ perspective will help to complement data previously collected by the New Brunswick Health Council on the patients’ perspective. These various perspectives may enhance interventions to help deal with the staffing shortage and assist policy decision-makers.

In our study, we aim to answer the following questions: How do the primary care models used in New Brunswick relate to the primary care providers’ preferences and do they influence job satisfaction levels? What other key factors drive satisfaction, and how can they be used to help with recruitment and retention? How are job satisfaction levels different for primary care providers working alone or in a team-based setting? How did the COVID-19 pandemic influence job satisfaction?

## Methodology

This research presents a descriptive and comparative study of primary care models in New Brunswick through data collected from primary care providers in the province. A total of 120 primary care providers participated in our study. The inclusion criteria for this project were individuals listed as family physicians or active nurse practitioners with privileges in one of the two Regional Health Authorities. As for the exclusion criteria, medical specialists, retired physicians, registered nurses who are not nurse practitioners in primary care, and those exempt from practicing in New Brunswick were not included.

### Setting

In New Brunswick, the province is divided into seven health zones (1 to 7), as shown in Table 1 below. In 2021, the New Brunswick Health Council gathered data from 13 500 patients in the province asking them where they access primary care [17, 19]. They found that depending on the health zone, patients were getting their care from different primary care models (Table 1). When comparing the percentages across the columns for “Family physician” primary care model, Zone 2 has a greater percentage of getting care from a family physician (66%) than in Zone 5 (51.8%). In contrast, fewer patients in Zone 2 reported getting their care from a Nurse practitioner (2.4%) than in Zone 5 (7.3%). Overall, Table 1 below presents the proportion of patients who reported getting their care from each primary care model (family physician, nurse practitioner, emergency department, after-hour/walk-in clinics or other).

**Table 1:** Primary care model usage per geographical zone in New Brunswick [17]

Primary care models in NB.	Zones in New Brunswick							
	**%**	**Zone 1**	**Zone 2**	**Zone 3**	**Zone 4**	**Zone 5**	**Zone 6**	**Zone 7**
Family physician	57	48.3	66	59.5	47.3	51.8	61	64.3
Nurse practitioner	3.9	2.4	4.6	4.9	2.5	7.3	3.5	5.0
Emergency department	10.4	7.4	10.7	9.9	26.5	16.6	9.3	7.3
After-hour/walk-in clinics	20.5	33.9	12.1	17.2	14.6	24.6	14.5	16.7
Other	12.1	10.4	11.2	13.4	11.6	7.3	15.2	11.7

### Data collection

Data collection took place over five weeks in March 2022. The list of family physicians contacted for this project was taken from the College of Physicians and Surgeons of New Brunswick’s website. This list was filtered to only include family physicians; it provided names and office or hospital telephone numbers. Research assistants called family physician offices directly to obtain consent. They sent the online survey link via email or the paper version by fax once they agreed to participate. The survey link and project summary were also published in the New Brunswick Medical Society e-bulletin. Nurse practitioners were recruited using the chain-referral sampling (or snowball sampling) method [20] using our networks and key individuals working in one of the two regional health authorities who could share the survey with their colleagues. This method was used since it was impossible to generate a list of nurse practitioners separate from all registered nurses in New Brunswick. The physician response rate is 13% for physicians (78 participants out of 600 physicians) and 34% for nurse practitioners (42 participants out of 124 nurse practitioners).

### Study sample

The study sample is presented in Table 2 below, where the primary care models reported by participants are divided by the geographical zone where they work. Similar to the data from the patient survey collected by the New Brunswick Health Council, our study also illustrates an uneven distribution of primary care models throughout the province. Table 2 shows that in zones 1 and 4, the proportion of physicians working in solo practice was higher than in the rest of the sample. Likewise, zone 3 and 4 have a higher proportion of participants working in collaborative practice.

**Table 2:** Primary care models reported by geographical zone

	N (%)								
Primary care model	**NB**	**Zone 1**	**Zone 2**	**Zone 3**	**Zone 4**	**Zone** **5**	**Zone** **6**	**Zone** **7**	
Physician in solo practice	33 (27.5)	11 (40.7)	8 (30.7)	8 (29.6)	5 (38.4)	0 (0)	1 (5.5)	0 (0)	
Physicians in collaborative practice	33 (27.5)	4 (14.8)	4 (15.3)	9 (33.3)	5 (38.4)	0 (0)	9 (50)		1 (16.6)
Collaborative practice physician and NP	27 (22.5)	5 (18.5)	5 (19.2)	8 (29.6)	3 (23)	2 (100)	3 (16.6)	1 (16.6)	
After-hours/walk-in clinics	2 (1.7)	1 (3.7)	1 (3.8)	0 (0)	0 (0)	0 (0)	0 (0)	0 (0)	
Community health center	13 (10.8)	1 (3.7)	5 (19.2)	2 (7.4)	0 (0)	0 (0)	2 (11.1)	2 (33.3)	
Emergency department	10 (8.3)	4 (14.8)	3 (11.5)	0 (0)	0 (0)	0 (0)	3 (16.6)	0 (0)	
Missing data	2 (1.7)	2 (7.4)	0 (0)	0 (0)	0 (0)	0 (0)	0 (0)	2 (33.3)	
Total	120 (100)	28 (23.3)	26 (21.6)	27 (22.5)	13 (10.8)	2 (1.6)	18 (15)	6 (5)	

Note. NP: nurse practitioner

### Data collection instrument

The NB Primary Health Care Access Survey used by authors Manuel et al. (in press) was also used to develop the survey used in our study [21]. It was adapted to include questions related to the New Brunswick primary care models. Specifically, we asked: Which statement most resembles the care model of the clinic where you work (an office with a family doctor in solo practice, an office with several family physicians in collaborative practice, a collaborative clinic with nurse practitioners, a collaborative clinic with other health professionals, an after-hours clinic or a walk-in clinic, a community health center, an emergency room or other?). We also asked questions to assess primary care providers’ satisfaction in their workplace: How do you describe your job satisfaction (responses were on a 5-point Likert scale: very dissatisfied, dissatisfied, neutral, satisfied, very satisfied). We asked about job satisfaction changes during the COVID-19 pandemic and symptoms of burnout. Regarding burnout, there was a survey question asking if the participant experienced the following symptoms: malaise, fatigue, frustrations, cynicism, or feeling of ineffectiveness. Using similar survey questions as authors Haggerty et al. (2004), this survey contains questions addressing the primary care provider profile (sex, years of work experience, job satisfaction, etc.) and queries relating to the organizational structure of specific primary care models (solo practice, collaborative physician team, collaborative nurse practitioner and physician team, or community-based clinics) [22]. Recruitment and retention factors that could influence primary care providers’ choices were outlined in the survey. Each participant was asked which factors were important to them at the time of recruitment (at the beginning of their career) and which factors are important to them now (retention) from a list of factors found in the literature review. The survey, written in French and English, consists of 31 questions. Although designed online using Survey Monkey, the survey was also available in paper format to accommodate primary care providers who preferred receiving it by fax to increase participation and statistical power.

### Data analysis

The data analysis was done using IBM’s “SPSS Statistics” software, version 28 [23]. The first part of the statistical analysis was descriptive. We used Chi-square and Fisher’s exact tests to compare the study variables to determine if there were statistically significant variations. The significance level for all tests was set at *p* < 0.05.

### Ethical approval and consent to participate

The research ethics committee at the Université de Moncton granted our study ethics approval and a reference registration number (file 2122-075).

## Results

Table 3 below is the description of our study sample. As shown in Tables 3 and 120 primary care providers completed the survey. Participants’ ages ranged from 30 to 74 years (data not shown), with an average age of 44 years for the sample. No significant difference in age was observed between physicians and nurse practitioners (*t* (111) = -0.504, *p* = 0.615), and no significant difference in gender was observed between provider types (chi-square [1] = 3.557, *p =* 0.059).

**Table 3:** Description of the study sample (n = 120)

Primary care provider	N	Mean age	N Sex (%)
Physicians	78	44 years	32 M (41) 46 F (59)
Nurse practitioners	42	43 years	10 M (24) 32 F (76)
Total	120	44 years	42 M (35) 78 F (65)

Note. M: male, F: female

Table 4 presents the reasons given for choosing their current primary care model. The primary care provider’s preference was the most popular reason reported. Nearly half of the sample reported choosing the primary care model based on their personal preference compared to 20.9% of providers who felt the decision was imposed on them by one of the regional health networks (19.2%) or the Department of Health (1.7%).

**Table 4:** Reasons reported for choosing their current primary care model

Reasons for choosing the current primary care model	%
Provider preference	45.8
Population health needs	22.5
Imposed by one of the regional health networks	19.2
Imposed by the Department of Health	1.7
Financial incentives	1.7
Other	9.1

Table 5 represents satisfaction levels, presented dichotomously by grouping the positive responses “satisfied” and “very satisfied” together. The results show a high satisfaction level among participants analyzed by current primary care models. Overall, 77% of participants responded as being satisfied. Satisfaction levels remained stable when compared between groups divided by primary care models. The results illustrate that satisfaction level does not significantly change for participants working in solo practice or collaborative practices, which means that job satisfaction levels remain stable regardless of the primary care model where participants worked.

**Table 5:** Job satisfaction level by primary care model

Primary care model		Unsatisfied n (%)	Satisfied n (%)	*p*
Physicians in solo practice	Yes	8 (30.8)	18 (69.2)	0.274
	No	15 (20.3)	59 (79.7)	
Physicians in collaborative practice	Yes	5 (18.5)	22 (81.5)	0.517
	No	18 (24.7)	55 (75.3)	
Collaborative MD and NP.	Yes	3 (11.5)	23 (88.5)	0.106
	No	20 (27.0)	54 (73.0)	
After-hours/walk-in clinics	Yes	2 (100)	0 (0)	0.051
	No	21 (21.4)	77 (78.6)	
Community Health Centers	Yes	2 (18.2)	9 (81.8)	0.999
	No	21 (23.6)	68 (76.4)	
Emergency department	Yes	3 (50.0)	3 (50.0)	0.133
	No	20 (21.3)	74 (78.7)	
Other	Yes	0 (0)	2 (100)	0.999
	No	23 (23.5)	75 (76.5)	
Total		23 (23)	77 (77)	

Note. The difference between satisfaction levels was the result of Chi-square tests when cell counts were all above 5 and Fisher’s exact testing when fewer than 5 counts in a cell

*Significance level p < 0.05

MD: medical doctor or physician

NP: nurse practitioner

Table 6 illustrates the reported changes in the job satisfaction levels of primary care providers during the COVID-19 pandemic. On this point, 55.2% of the participants expressed a deterioration in their job satisfaction. However, no significant difference was observed between providers based on the primary care model. The summary table below presents the data on how the changes to job satisfaction levels during the COVID-19 pandemic were not significantly different between primary care providers working in solo and collaborative practice.

**Table 6﻿:** Changes to job satisfaction during the COVID-19 pandemic based on the primary care model

Primary care model	Improvement n (%)	Stable n (%)	Deterioration n (%)	*p*
**Providers in solo care**	0 (0)	14 (50.0)	14 (50.0)	0.260
**Providers in collaborative care**	4 (5.9)	25 (36.8)	39 (57.4)	
**Total**	4 (4.1)	39 (41.0)	53 (55.2)	

Note. The difference in satisfaction levels was determined by Chi-Square testing

* Significance level p < 0.05

Table 7 presents the proportion of participants who reported symptoms of burnout. This analysis found that 53.3% of the care providers reported having felt at least one symptom of burnout. The proportion of providers who reported burnout symptoms did not vary significantly based on their primary care model.

**Table 7:** Symptoms of burnout reported by participants based on their primary care model

		Symptoms of burnout		
Primary care model		**Yes** **n (%)**	**No** **n (%)**	***p***
Physician in solo practice	Yes	15 (50.0)	15 (50.0)	0.672
	No	42 (54.5)	35 (45.5)	
Physicians in collaborative practice	Yes	17 (60.7)	11 (39.3)	0.358
	No	40 (50.6)	39 (49.4)	
Collaborative MD and NP.	Yes	11 (40.7)	16 (59.3)	0.131
	No	46 (57.5)	34 (42.5)	
Afterhours/walk-in clinics	Yes	1 (100)	0 (0)	0.999
	No	56 (52.8)	50 (47.2)	
Community Health Centers	Yes	6 (60.0)	4 (40.0)	0.654
	No	51 (52.6)	46 (47.4)	
Emergency department	Yes	5 (55.6)	4 (44.4)	0.886
	No	52 (53.1)	46 (46.9)	
Other	Yes	2 (100)	0 (0)	0.498
	No	55 (52.9)	49 (47.1)	
Total		57 (53.3)	50 (46.7)	

Note. The difference between satisfaction levels was the result of Chi-square tests when cell counts were all above 5 and Fisher’s exact testing when fewer than 5 counts in a cell.*Significance level *p* < 0.05

MD: medical doctor or physician NP: nurse practitioner

In total, 15 recruitment and retention factors, potentially deemed influential to the primary care providers’ choices when deciding where to work, were presented as options in the survey. Each participant was asked which factors were important to them at the time of recruitment (at the beginning of their career) and which factors are important to them now (retention). Table 8 presents the factors with the highest proportion of participants who chose each option related to recruitment. Most factors did not vary significantly between family physicians and nurse practitioners. There was the exception for the opportunity to work in multiple environments and remuneration that were more important recruitment factors (*p* 0.006) for family physicians.

**Table 8﻿:** Proportion of participants by reported recruitment factors (n = 119)

Recruitment factors	MD n (%)	NP n (%)	Total n (%)	*p*
Proximity to family and friends	30 (39)	12 (29)	42 (35)	0.257
Work-family balance	29 (38)	10 (24)	39 (33)	0.124
Professional autonomy	21 (27)	17 (40)	38 (32)	0.140
Living conditions	20 (26)	5 (12)	25 (21)	0.072
Professional support	14 (18)	10 (24)	24 (20)	0.465
Remuneration	15 (19)	2 (5)	17 (14)	0.028*
Opportunity to work in multiple environments	20 (26)	2 (5)	22 (19)	0.004*
Nearby infrastructure	12 (16)	5 (12)	17 (14)	0.584
An internship in the region	8 (10)	3 (7)	11 (9)	0.559
Total participants	77 (65)	42 (35)	119 (100)	

Note. The differences were determined by Chi-Square testing tests when cell counts were all above 5 and Fisher’s exact testing when fewer than 5 counts in a cell

* Significance level *p* < 0.05. MD : Family physician NP: Nurse practitioner

Table 9 presents the same information as Table 8, but for factors related to retention. All factors were similar between family physicians and nurse practitioners with the exception of living conditions was a more important retention factor for family physicians (*p =* 0.004).

**Table 9﻿:** Proportion of participants by reported retention factors (n = 119)

Retention factors	MD n (%)		NP n (%)	Total n (%)	*p*
Proximity to family and friends	37 (48)		14 (33)	51 (43)	0.121
Work-family balance	38 (49)	13 (31)		51 (43)	0.053
Professional autonomy	21 (27)		16 (38)	37 (31)	0.223
Living conditions	34 (44)		7 (17)	41 (35)	0.003*
Model of primary care	17 (22)		4 (10)	21 (18)	0.086
Remuneration	8 (10)		5 (12)	13 (11)	0.800
Nearby infrastructure	8 (10)		3 (7)	11 (9)	0.559
Access to continuing education	5 (6)		5 (12)	10 (8)	0.309
Financial incentives	6 (8)		3 (7)	9 (8)	0.898
Total	77 (65)		42 (35)	119 (100)	

Note. The differences were determined by Chi-Square testing tests when cell counts were all above 5 and Fisher’s exact testing when fewer than 5 counts in a cell

* Significance level *p* < 0.05. MD : Family physician NP: Nurse practitioner

Tables 10 and 11 present the comparative analysis between family physicians and nurse practitioners for key satisfaction indicators. There are no statistically significant differences between types of primary care providers.

**Table 10:** Comparative analysis between physicians and nurse practitioners for satisfaction and exhaustion indicators

	Yes n (%)	No n (%)	*p*
**Burnout**			
Physician	34 (49.3)	35 (50.7)	0.31
Nurse practitioners	23 (60.5)	15 (39.5)	
Total	57 (53.3)	50 (46.7)	
**Job satisfaction**			
Physician	20 (25.6)	58 (74.4)	0.29
Nurse practitioners	15 (35.7)	27 (64.3)	
Total	35 (29.2)	85 (70.8)	

Note. The differences were determined by Chi-Square testing tests when cell counts were all above 5 and Fisher’s exact testing when fewer than 5 counts in a cell

* Significance level *p* < 0.05

**Table 11﻿:** Comparative analysis between physicians and nurse practitioners concerning changes during the COVID-19 pandemic

	Improve n (%)	Stable n (%)	Deterioration n (%)	*p*
Physician	2 (3.0)	25 (37.3)	40 (60)	0.52
Nurse practitioners	3 (7.3)	16 (39)	22 (53.7)	
Total	5 (4.6)	41 (38)	62 (57.4)	

Note. The differences were determined by Chi-Square testing tests when cell counts were all above 5 and Fisher’s exact testing when fewer than 5 counts in a cell. * Significance level *p* < 0.05

## Discussion

The main finding of our study was that a substantial proportion (77%) of participants declared being satisfied at work by expressing a moderate to high level of job satisfaction. The reported job satisfaction levels did not appear to be influenced by the primary care model. As such, primary care providers reported similar job satisfaction levels regardless of the primary care model or whether they worked in collaborative or solo practice. The findings suggest that irrespective of the model of care, what is important to providers is having the professional autonomy to choose the care model that best suits their individual preferences. Weber (2015) agrees with this finding, stating the decision-making autonomy of physicians is a determining factor in their professional satisfaction [24]. Most physicians agree on the importance of autonomy, and add that having control over their daily practice is an important predictor of job satisfaction [25, 26]. This also means having autonomy over their schedule, and the choice to increase their patient load, which is associated with a higher level of satisfaction than if this load is imposed [25]. However, sometimes to control outcomes or performance, some decision-makers or government officials may set new policies that can reduce their autonomy. Consequently, such initiatives may reduce their job satisfaction [27]. In addition, administrative overload, evaluation standards, and time pressures also tend to lower their level of satisfaction (Bovier & Perneger, 2003; Epstein, 2000; Weber, 2015). It is important to reflect on the risk-benefit analysis of imposing specific standards on physicians (on patient load, for example) as it may influence autonomy, and then may impact job satisfaction. Generally, a heavy-handed or authoritarian approach may backfire, and it is crucial to include primary care professionals on topics that will influence their practice. For example, imposing a minimum number of patients in their roster or that a certain number of patients are seen per day, requiring that primary care providers work in collaboration, or imposing a specific pay structure, are all situations that could limit their autonomy. Although the benefits of measuring outcomes or performance are also important, they should be done in a way that promotes the empowerment and autonomy of primary care providers.

The potential changes to job satisfaction levels with the COVID-19 pandemic must also be considered. According to the results of our study, 55.2% of the care providers in the sample expressed a decline in their level of satisfaction since the pandemic. In contrast, only 5% indicated an improvement. Some researchers confirm that physicians are vulnerable to dissatisfaction and burnout symptoms, as was established by a group of physicians in a paper on the subject published during the COVID-19 pandemic [28]. The findings of our study suggest that the reported deterioration in job satisfaction was associated with factors other than models of care since there was no significant difference in changes to job satisfaction between providers who work alone and or as part of a team. This suggests that other factors, such as decreased direct contact with patients during the COVID-19 pandemic, may explain the reported deterioration in satisfaction [24]. In a study out of New Brunswick on the use of telehealth during the COVID-19 pandemic, many physicians reported seeing the benefit of incorporating telehealth into their practice [29]. Still, they also said telehealth was overused during the pandemic [29]. The perceived overuse of telehealth was a source of anxiety for them since they worried about the health and well-being of their patients. In addition, the changes to their practice brought on by the COVID-19 pandemic left them little time to get organized. The urgency of the changes brought to their practice also impacted their level of job satisfaction [29]. At this time, it is difficult to determine whether the deterioration observed in the results is indeed linked to personal stressors, which generally affected the population during the COVID-19 pandemic, or to factors directly related to work. Further studies will undoubtedly provide clarity on this critical topic in the future.

More than half (53.3%) of primary care providers surveyed revealed that they had experienced one or more symptoms related to burnout. The proportion found in our study is similar to results from the United States, where roughly 50% of physicians reported signs of burnout before the COVID-19 pandemic [30]. Furthermore, participants reported no significant difference in burnout symptoms based on their models of care, once again illustrating that working alone or in a team does not appear to be a determinant in predicting burnout symptoms. This indicator should be carefully considered, as burnout can have severe consequences for the personal well-being of the person experiencing it, as well as a deterioration in job satisfaction, the care provided and employee retention [30, 31]. Healthcare provider burnout is often linked to excessive workloads, inefficient work processes, administrative burdens, work-home conflicts and lack of contribution or control [31]. Although mostly anecdotal at this time, there have been multiple media reports on how the COVID-19 pandemic has added strain on healthcare providers, which has increased the risk of developing burnout symptoms, often leading to an increase in turnover rates and worsening staff shortages [3, 32]. Ultimately, this creates a vicious cycle, where healthcare providers end up working with fewer resources (or “short staffed”); this increases workload, which increases symptoms of burnout and leads to people leaving their jobs, and once again leads to more staff shortages [30]. Through primarily anecdotal reports, it is reasonable to think that the COVID-19 pandemic has contributed to an increase in burnout symptoms. It may explain why many healthcare providers leave their jobs or professions [33]. Given the exogenous shocks brought on by the COVID-19 pandemic that impacted an already fragile workforce, strategies to retain and recruit healthcare providers in New Brunswick have become a priority for leaders and government officials.

Recruitment and retention success depends on proximity to family and friends, work-family balance and professional autonomy according to the results of our study. They were reported as predominant at the time of career establishment (recruitment) and at the time of survey (retention). Multiple studies support the importance of community factors (like proximity to family and friends) as an important determinant for recruitment and retention [34–36]. In addition to proximity to family and friends (ranked first, 35% of respondents reported it as important during recruitment and 43% for retention), reconciling work and family life was also a priority, ranking second in the rate of responses obtained with 33% of respondents reporting it as important during recruitment, and 43% of them stating it was important for retention. Johnson & Ravitsky (2015) studied how work-family balance was challenging for physicians (especially women) with children. Consequently, the lack of balance can harm physician satisfaction (especially for physicians 35 to 54 years old) [37]. In multiple studies, work-life or work-family balance strongly predicts physicians’ job satisfaction [26, 38–40]. In partnership with Family Medicine New Brunswick, the province of New Brunswick has considered this by promoting the development of family medicine units that offer the advantage of work-family balance [41]. Remarkably, the top four most frequently chosen factors during recruitment (proximity to family and friends, work-family balance, professional autonomy and living conditions) were the same factors chosen as important during retention. However, most studies on recruitment and retention tend to highlight the differences between the two strategies [34]. Based on the findings of our study, the same factors seem to be important throughout primary care providers’ careers, which could make recruitment and retention strategies easier in the province, since the factors may remain the same. Overall, there was little variation between factors important during recruitment or retention for family physicians and nurse practitioners. The discrepancies between recruitment and retention for different groups of professionals should be examined in more depth in future studies to understand how to utilize them more efficiently.

As with determining patient load and job satisfaction, professional autonomy emerges as a crucial element for recruitment for family physicians and nurse practitioners. Approximately 30% of participants consider it important when establishing their practice and staying in their job. In addition to being a springboard for satisfaction, professional autonomy is important for the doctor who wants to practice freely without too many constraints (Koebisch et al., 2020). The autonomy to choose the type of primary care model may explain many of our study’s findings since almost half of the participants reported choosing their current primary care model based on preference. In addition, this phenomenon was supported by the minimal variations observed between primary care providers working alone or as part of a team. Many participants shared in the survey’s written comments section (data not shown) that they sought a practice that suited them when establishing their careers. They shared that having the autonomy to choose the location, the type of primary care model and the size of their practice was especially important to them. However, a few participants reported not feeling very empowered at the beginning of their careers. Those participants stated that they took over an established practice in a desirable geographical location and kept it running in a similar fashion. Many participants did not feel compelled to modernize the practice they took over. At the same time, others described taking over an established practice, then changing it to match their preferences.

It was surprising to note that financial incentives are not considered an important recruiting factor and constitute an attractive factor for retention for only a few participants (8%). Although many studies on recruitment and retention report financial incentives as not being a strong predictor [34, 39, 42, 43], it remains a popular government strategy (signing bonuses and loan repayments, for example) [13, 44]. According to some of these same authors, it would be preferable to use financial incentives strategically. For instance, if they were granted in the form of a support program for partners’ relocation or integration into the community [34]. That way, the financial incentive would encompass other factors that could help the primary care provider (and their family) integrate into the community, which is more important for predicting retention [45]. Focused and strategically placed financial incentives may help recruit and retain primary care providers more effectively.

Finally, nurse practitioners were recruited as primary care providers for our study because they are often targeted as part of the solution to improve access to primary care in Canada [46]. The findings in Tables 10 and 11 illustrated how little variations were observed between nurse practitioners and family physicians for key satisfaction indicators during our study. However, it is difficult to compare with other studies on job satisfaction and retention of nurse practitioners in primary care because of the lack of data on the subject. Further studies are needed to better understand the differences between nurse practitioners and physicians in primary care. Currently, nurse practitioners cannot open a solo practice to offer medical services covered by public insurance because they are required to work with a physician in New Brunswick. However, nurse practitioners reported having many options when choosing where to work, in other words there are various fields where they can practice [47]. In our study, nurse practitioners reported that having many options to choose from when deciding where to work was important to them. But, once a nurse practitioner chooses a work place, it is not as crucial to them to have the opportunity to work in multiple environments. Our results highlighted the importance for nurse practitioners in primary to have professional autonomy, this is a topic that needs a more in-depth investigation.

## Limitations

This observational study does not allow us to conclude causal links between the various variables analyzed or allow for generalizability because of the small sample. Thus, the study can only present the findings from the perspective of primary care providers. Lastly, the results of this study were self-reported with some qualitative data as part of the survey, which may include social desirability bias and missed nuances due to the inherent limitation with an online survey.

## Conclusion

In closing, the main findings from this study are that primary care providers reported similar job satisfaction levels among providers who practice alone or in collaboration. Similarly, of the roughly 50% of primary care providers who reported having symptoms of burnout and a decline in job satisfaction during the COVID-19 pandemic, the proportions were similar between providers who worked alone compared to those who worked in collaborative practice. This suggests that the primary care model did not seem to be linked to job satisfaction, symptoms of burnout or deterioration of satisfaction during the COVID-19 pandemic. However, the opportunity to choose a primary care model that suits the providers’ preferences seemed to be more important than a specific primary care model. In other words, physicians’ autonomy to choose appears to be a key determining factor in their job satisfaction levels. Which, in turn, could increase retention.

The findings in our study suggest that primary care models may be moderately important for retention, whereas proximity to family and friends and balancing work and family emerge as critical factors that influence choosing a job (recruitment) and staying in that job (retention). These factors should be included in primary care providers’ staffing recruitment and retention strategies. Furthermore, in future research, a more in-depth examination of the drop in the level of satisfaction linked to the COVID-19 pandemic could help understand how a crisis can impact job satisfaction. The information could guide recruitment and retention strategies to support primary care providers during trying times.

## Data Availability

The dataset generated during the current study is not available in a publicly accessible repository because the Université de Moncton does not have a place to store datasets. However, the dataset used could be available from the corresponding author upon reasonable request.
